# Prevalence of occupational injury and associated factors among building construction workers in Dessie town, Northeast Ethiopia; 2018

**DOI:** 10.1186/s13104-019-4436-4

**Published:** 2019-08-05

**Authors:** Teferi Gebru Gebremeskel, Tesfaye Yimer

**Affiliations:** 1 Department of Reproductive Health, College of Health Sciences, Aksum University, Aksum, Ethiopia; 2 Department of Public Health in Occupational Health and Safety Management, College of Health Medicine and Sciences, Gondar University, Gondar, Ethiopia

**Keywords:** Prevalence, Occupational injury, Building constructions workers

## Abstract

**Objective:**

This study aimed to assess the prevalence of occupational injury and associated factors among building construction workers in Dessie town, Northeast Ethiopia.

**Result:**

The prevalence of occupational injury was 32.6% 95% CI (27.8–37.4). Male workers [AOR: 1.775, 95% CI (1.108–2.844)], uneducated [AOR: 3.327, 95% CI (1.262–8.771)], training [AOR: 2.053, 95% CI (1.004–4.195)] and Uses of PPE [AOR: 2.076, 95% CI (1.253–3.439)]. In focus group discussions negligence of the workers and lack of awareness were factors significantly associated with Occupational injury. The occupational injury was high among construction workers. Sex, Educational status, Safety training, Personal protective equipment were statically significantly associated.

**Electronic supplementary material:**

The online version of this article (10.1186/s13104-019-4436-4) contains supplementary material, which is available to authorized users.

## Introduction

Globally, hundreds of millions of people are working in unsafe conditions [1, 2]. ILO recently estimated that globally about 2.2 million people die every year [3]. The economic cost of work-related injury and illness varies between 1.8 and 6% GDP in a country and an average of 4% GDP globally in 2016 [1, 2]. The impact of occupational health and safety hazards faced by construction workers in developing countries is 10 to 20 times higher than those in industrialized countries [1, 2]. In Ethiopia, building construction industries are the second leading cause of injury next to a traffic accident and the risk of fatality in the construction sector is five times higher than in manufacturing industries [4]. Prevalence of occupational injuries among construction workers was in Egypt 46.2% [5], in Kenya 74% [6] and in Ethiopia, the prevalence varied between 38.3 and 84.7% [7, 8]. Factors contributing to these prevalence rates of occupational injuries were lack of safety training, job stress, the absence of safety signs, sleep problems, workload, drinking alcohol, and chewing khat [6–8]. If urgent interventions are not in place, occupational injuries and loss of productivity will continue to be a major challenge in the construction industry in the future [8]. The findings of this study were providing a prevention strategy designed for occupational injuries among construction workers and will be helpful for planners and policymakers to design intervention and appropriate control methods in the workplace.

## Main text

### Methods

#### Study setting and design

Institutional-based cross-sectional quantitative study triangulated with a qualitative approach was conducted from March to April 2018 in Dessie town, North-east of Ethiopia. The total number of workers in building construction industries was 1200 in different working professions. All workers directly involved in the process of construction in the last 1 year and labor inspectors were included while, administrative workers, supportive staffs and minors were excluded.

#### Study sampling

A single population proportion formula was used to calculate the quantitative sample size, by using a 38.7% prevalence of occupational injury among construction workers [9]. For qualitative, three FGD that each consisted of 6 to 7 homogeneous participants were used.

For Quantitative data, Simple random sampling technique was used to select the study participants. The study populations were proportionally allocated from all private construction industries. Study participants from each construction industries were drawn by computer-generated random number method and three FGD were used to collect qualitative data to explore health and safety system of building construction industries. Purposive sampling techniques were used.

#### Data collection tool

Quantitative data were collected using pre-tested and interview administered questioner. Four-degree graduate and two supervisors are MPH graduate conducted the data collection process.

A total of three FGD were used to collect Qualitative data to describe the health and safety system of building construction industries to reach the level of saturation. The qualitative data were collected by using a tape recorder and note taking method after obtaining verbal consent from participants.

### Data management and analysis

The data was entered, edited and organized first using EPI info version 3.5.4 and then exported SPSS 20 for analysis. All variables with p ≤ 0.2 in the bivariate variable analysis were inserted in the multivariable logistic regression model. Binary Logistic regression was used to find the association between the characteristics of the independent variable and the dependent variable p < 0.05 was considered as significant throughout the study. The transcribed documents were imported into Atlas.ti version for qualitative data analysis software version 7 for coding and analysis. The investigators then coded the respondent’s phrases, sentences, and memos that were relevant to the area of the study. They systematically coded raw data openly and categorized the sub-themes under their respective themes. Then, they created non-repetitive central themes that were constructed based on the natural meaning of categories. An inductive approach was followed to allow the conceptual cluster of ideas and patterns to emerge.

### Results

#### Socio-demographic characteristics

Of the total 402 questionnaires distributed, 374 fully completed, giving a response rate of 93.03%. Prevalence of occupational injury in the last 12 month was 32.6% 95% CI (27.8–37.4). The mean age of the respondents was 25.69 (SD ± 6.664) years (Table 1).

**Table 1:** Socio demographic characteristics of the study participants in Dessie town building construction workers, Northeast Ethiopia, 2018 (n = 374)

Variable	Number	Percent
Sex		
Male	193	51.6
Female	181	48.4
Marital status		
Married	130	34.8
Single	244	65.2
Employment condition		
Permanent	96	25.7
Temporarily	278	74.3
Work experience (years)		
1–5	313	83.7
≥ 6	61	16.3
Age (years)		
< 18	188	50.3
18–24	129	34.5
25–49	31	8.3
≥ 50	26	7
Educational status		
Uneducated	82	21.9
Primary school (1–8)	160	42.8
Secondary school (9–12)	99	26.5
Diploma and above	33	8.8
Working profession		
Mason	48	12.8
Carpenter and roofers	37	9.9
Plumber and electricians/welders	22	5.9
Plasterer	19	5.1
Daily laborers and other helpers	223	59.6
Operators/drivers	25	6.7

#### Working environmental factors

From the participants, only 63 (16.8%) respondents were receiving health and safety training. This finding also supplemented by qualitative from the FGD Quoted as follow:

A respondent from the worker’s group discussions said*: “no one gives much concern about safety also employers are not interested to give health and safety training to us for the reason to keep their time and protect them from undesired payment (WFGDID4, age 25)”.*

Similarly, Participants from the Contractors group discussions said *“Employers commencement of their work without giving pre*-*work training for employees (CFGD1, age 28)”.*

Participants from the Government stakeholder group discussions *said “Employers commence their work before they give training about occupational safety & health (GFGDID2, age 33)”* (Table 2).

**Table 2:** Working environmental and behavioral factors of the study participants in Dessie town building construction workers, Northeast Ethiopia, 2018 (n = 374)

Variable	Number	Percent
Regular health and safety supervision		
Yes	57	15.2
No	317	84.8
Hours working per day (hours)		
≤ 8	309	82.6
> 8	65	17.4
Hours working per week (hours)		
≤ 48	301	80.5
> 48	73	19.5
Health and safety training		
Yes	63	16.8
No	311	83.2
Utilization of PPE		
Yes	147	39.3
No	227	60.7
Smoking cigarette		
Yes	77	20.6
No	297	79.4
Alcohol user		
Yes	130	34.8
No	244	65.2
Khat chewing		
Yes	99	26.5
No	275	73.5

#### Behavioral characteristics

From the total respondents, 147 (39.3%) utilize PPE on duty from these 52 (35.4%) were used Helmet and 43 (29.3%) were used Gloves. This finding also supplemented by qualitative from the FGD Quoted as follow:

Workers are said *“we are not using safety materials, the main reasons are due to financial scarcity most organization buy safety needs and materials for permanent workers (WFGDID7, age 23)”.*

From Contractors, FGD participants said*, “Workers in construction industries are not using safety materials, the main thing here is that the cost of safety materials are expensive because of this the construction industry do not provide enough materials (CFGDID5, age 29)”.*

In the same way, other governmental stakeholders said *“… In many construction industries there is a tendency of not providing PPE by the employer for their workers. But in some construction industries, the employers provide PPE for their workers, but the workers don’t use PPE’s due to doesn’t get pre*-*work training about PPE” (GFGD ID4, age 24).*

Similarly, other members of the group said*: “many of the workers do not use PPE Because of the workers PPE‘s doesn’t much with the work, and it is size not much with the worker’s body (GFGDID1, age 27)”.*

In the other 22 years governmental stakeholders, ID3 said*: “due to lack of awareness 90% of workers do not use safety materials at their working place”* (Table 2).

#### Factors associated with injuries in building construction

Bivariate analysis showed that Sex, Educational status, utilization of PPE, receiving health and safety training, alcohol drinking, Cigarette smoking, and Chewing Khats were found to be significantly associated with occupational injury.

In multivariable logistic regression, sex, Educational status, utilization of PPE and health and safety training were statistically significant associated with occupational injury.

Among the respondents, the odds of Occupational injury among male workers were 1.7 times more than compared with female workers [AOR: 1.775, 95% CI (1.108–2.844)].

The odds of occupational injury were 3.327 (95% CI 1.262, 8.771) times higher among uneducated workers as compared to educated workers holding other variables constant. Among the participants, the odds of occupational injury who didn’t receive health and safety training were about 2 times higher than those who were receiving health and safety training [AOR: 2.053, 95% CI (1.004–4.195)].

The odds of occupational injury were 2.076 (95% CI 1.253, 3.439) times higher among workers who did not use PPE as compared to workers who use PPE holding other variables constant (Table 3).

**Table 3:** Factor associated with occupational injury among building construction workers in Dessie town by bivariate and multivariable logistic regression (n = 374)

Variable	Occupational injury		COR (95% CI)	AOR (95% CI)
	Yes	No		
Sex				
Male	73	120	*1.639 (1.057*–2.540)*	*1.775 (1.108*–2.844)*
Female	49	132	*1*	*1*
Educational status				
Uneducated	39	43	*2.834 (1.145*–7.016)*	*3.327 (1.262*–8.771)*
Primary school	47	113	1.300 (0.547–3.0895)	1.135 (0.453–2.845)
Secondary school	28	71	1.232 (0.497–3.057)	1.243 (0.475–3.253)
Diploma and above	8	25	*1*	*1*
Safety training				
Yes	12	51	*1*	*1*
No	110	201	*2.326 (2.296*–5.729)*	*2.053 (1.004*–4.195)*
PPE utilization				
Yes	35	112	*1*	*1*
No	87	140	*1.989 (1.250*–3.164)*	*2.076 (1.253*–3.439)*
Smoking cigarette				
Yes	33	44	*1.753 (1.047*–2.934)*	1.655 (0.937–2.923)
No	89	208	*1*	*1*
Alcohol users				
Yes	50	80	*1.493 (1.051*–2.336)*	1.454 (0.891–2.373)
No	72	172	*1*	*1*
Khat chewers				
Yes	41	58	*1.693 (1.051*–2.727)*	1.597 (0.947–2.694)
No	81	194	*1*	*1*

* p < 0.05, reference = 1.00

### Discussion

The main objective of this study was to assess the prevalence of occupational injury and its associated factors among building construction workers in Dessie town. In this study, the overall level of Occupational injury was 32.6%. The result is in line with the study conducted in Addis Ababa and Uganda i.e. 34.6% [10] and 32.4% [11] respectively. This consistency might be due to the prevention strategies and economic development of the countries. However the prevalence rate in this study was lower than a study conducted in Kancheepuram district, Tamil Nadu 44.3% [12], Egypt 46.2% [13]. This discrepancy might be due to the difference in working condition, as well as difference socio-cultural and regulatory factors adopted by concerned states, Besides, there is the difference in the study setting and design between other studies done and this study.

Studies were done in Ethiopia, Addis Ababa 38.3% [8], 84.7% [7] and in Gondar 38.3% [9] were higher prevalence reported. This difference might be the difference in study working conditions, levels of accident prevention strategies and there is a difference in study setting and time between other studies done and this study.

Among the respondents the odds of occupational injuries among male workers were two times higher as compared to female workers. The result is consistent with the study conducted in Gondar [9], Namibia [14], Tanzania [15], China [16] and USA [17]. This consistency may be due to male workers were highly exposed to occupational injury because males commonly do harder tasks leading to injuries [9], female workers used PPE more often than male workers [18], as well as male workers, are usually exposed to certain substances.

As per this study, the odds of Occupational injuries among uneducated workers were three times higher as compared to educated workers. This result was in line with the study conducted in Addis Ababa, Assessment of Municipal waste materials collector workers [10]. This consistency might be Higher education May provide the skills and knowledge about the means of protecting themselves from injury with the increasing levels of education, unsafe actions are reduced. High rates of unsafe actions among people with low literacy might be due to low levels of knowledge and lack of awareness about unsafe actions and being given difficult and dangerous tasks [19] and educated workers were work in safe working professions. In this study, the result showed that the odds of occupational injury who didn’t receive occupational health and safety training were 2 times more than workers who receive health and safety training. This result is in line with the study conducted in Gondar [9], Kenya [20], Ilam (Western Iran) [21]. This similarity might be workers participate in health and safety training increases their awareness and protect themselves from injuries. Similarly findings from qualitative approach supported that; From the government FGD participants *said “during the time of inspection majority workers who didn’t receive Occupational health and safety training in construction industries were injured GFGDID1, age 27)”* From the contractor’s discussion a participant supported *“Workers commencement of their work without getting enough training, this might be the cause of the problem (occupational injury)” (CFGDID1, age 28)* Participants from the Government stakeholder FGD *said “Employers commence their work before they give training about occupational safety & health (GFGDID2, age 33).* In this study, the odds of occupational injury were two times higher among workers who did not use PPE as compared to workers who use PPE. These results were consistent with the study done in Addis Ababa [7, 8], Nairobi, Kenya [20]. This consistency might be didn’t use PPE was no provision of PPE from the employers, negligence of workers and PPE were discomfort for the working condition of workers. Findings from qualitative FGD were supported; An individual from workers FGD said: “we are not using safety materials due to financial scarcity most organizations buy safety needs and materials for permanent workers only (WFGD ID7, age 23)”. Similarly “… due to a lack of occupational health and safety materials, we are more exposed to Occupational Injuries. (WFGD ID5, age 26)”. Respondents from Contractors discussions said, “The main thing here is that costs of safety materials are more expensive because of this we do not provide enough materials for workers (CFGDID5, age 29)”. Similarly, other members of the group said*: “many of the workers do not use PPE Because of the workers PPE‘s doesn’t much with the work, and it is size not much with the worker’s body (GFGDID1, age 27)”.*

In the other 22 years of governmental stakeholders, ID3 said *“due to lack of awareness 90% of workers do not use safety materials at their working place.*

### Conclusion

This study showed that the magnitude of Occupational injury was high (32.6%). It was lower than when we compared with other studies. Sex, Educational status, Utilization of PPE and safety training in both quantitative and qualitative studies were factors for injury in building construction workers.

## Limitation of the study

The studies were conducted 12-month prevalence of injury it might recall biases.

## Additional file

**Additional file 1.** Minimal data set used in the manuscript.

## Data Availability

The datasets used during the current study available from the corresponding author on reasonable request (Additional file 1).

## Abbreviations

AOR: adjusted odds ratio
BOLSA: Bureau of Labor and Social Affairs
CI: confidence interval
COR: crude odd ratio
ETB: Ethiopian Birr
FGD: focus group discussions
GDP: gross domestic product
ILO: International Labor Organization
OSH: Occupational Safety and Health
PPE: Personal protective equipment
SD: standard deviation
SPSS: Statistical Package for Social Science
UOG: University of Gondar
USA: United State of America
VIF: variable inflation point
