# Supplementary material for: Prevalence of occupational injury and associated factors among building construction workers in Dessie town, Northeast Ethiopia; 2018
Source: BMC Res Notes. 2019 Aug 5;12:481. doi: 10.1186/s13104-019-4436-4 (PMC6683456; doi:10.1186/s13104-019-4436-4)
Supplement: Supplementary file 1 — Additional file 1. Minimal data set used in the manuscript. [file 13104_2019_4436_MOESM1_ESM.docx]

## Annex- 1. English version consent form

University of Gondar

College of Medicine and Health Sciences

Institute of Public Health

Questionnaire for Prevalence of Occupational injury and associated factors among building construction workers in Dessie town, North east Ethiopia 2018

Verbal consent form before conducting interview

Greeting

Hello, I am__________________. I am working in the research team of school of Public Health College of medicine and health science, Gondar University. I would like to ask you a few questions about prevalence of injury. This will help to improve occupational safety, health and working environment services provided to you based on your answer to our questions. Your name will not be written in this form and will never be used in connection with any information you tell us. All information given by you will be kept strictly confidential. Your participation is voluntary and you are not obliged to answer any question you do not wish to answer. If you fill discomfort with the interview please fill free to drop it any time you want. This interview will take about 30 minutes. Do I have your permission to continue?

1 yes ---------- 2 No ---------

Informed consent certified by

Interviewer Code ___________ name ________ signature ____________

Date of interview _______ Time started ________ time completed --------result interview 1.Completed -----

2. Respondent not available -----

3. Refused ---------

Supervisor: Name ___________________signature ___________Date ______

| SN | Questions | Possible Response | Skips |
| --- | --- | --- | --- |
| Q101 | Sex | 1. Male 2 female |  |
| Q102 | Age | --------- year |  |
| Q103 | Marital status | 1. Single 2.Married 3.Divorce 4.Widowed 5.Others --------- |  |
| Q104 | Education status | 1. Uneducated 2. Read and write 3. Primary education 4. Secondary education 5. Diploma 6. degree and above |  |
| Q105 | Employment pattern | 1. Temporally 2. Permanent |  |
| Q106 | Year of experience | ---------year |  |
| Q107 | What is your working profession? | 1. Mason 2.Carpenter & roofers 3. Plumbers & electrician /welder/ 4. Worker carrying out finishing work /painter/ 5. Plasterer 6. Daily laborer and other helpers 7. Operator/driver |  |
| Q108 | Year of experience in current job | ------------------ |  |
| Q109 | Monthly income | ------------------------------ETB (birr) |  |

## Annex- 2- English version questionnaires

Part I: Socio demographic characteristics

Questionnaire ID ------------

**Part II Availability and utilization of personal protective measure**

Questionnaire ID ----------------------

| SN | Questions | Possible Response | Skips |
| --- | --- | --- | --- |
| Q201 | Do you use any PPE while you are on work? | 1. Yes 2. No | If no Skip to Q204 |
| Q202 | If yes to Q201 what type?(more than one answer is possible) | 1. Glove 2. Ear plug 3. Respirators  4. Helmet 5. Overalls 6. Goggles  7. Face shield 8. Boots /shoes  9. Others, specify |  |
| Q203 | From where do you get PPE? (more than one answer is possible) | 1. It is supplied by institution 2. You buy it for yourself 88. Others, specify |  |
| Q204 | If No to Q201, what are the reasons not use safety equipments? (more than one answer is possible) | 1. Feel discomfort 2. To save time 3.Not aware of risk 4.careless/negligence  5. No access 6. Don’t know its use/importance |  |
| Q205 | Have you ever had on job training on any type of occupational safety issues? | 1.Yes  2.No |  |
| Q206 | If yes to Q14, from where did you get? (more than one answer is possible) | 1. From institution 2.From Gov’t 2. From NGOs 3. Others--------- |  |

**Part III occupational (work related) injury characteristics**

Questionnaire ID ----------------------

| SN | Questions | Possible Response | Skips |
| --- | --- | --- | --- |
| Q301 | Have you had an accident at work that resulted injury to you in the last 12 months | 1. Yes  2. No | If no Skip to part IV |
| Q302 | Parts of the body affected | -------------------- |  |
| Q303 | Types of injury | ------------------ |  |
| Q304 | What was your reason(s) at, the time of injury? | -------------------------- |  |
| Q305 | How long it takes? | ……….. days |  |
| Q306 | Days of injuries | ------------ |  |
| Q307 | Time of injuries | ------------ |  |
| Q308 | Days lost | ----------- days |  |

**Part -IV-Working environment related variables**

Questionnaire ID ----------------------

| SN | Questions | Possible Response | Skips |
| --- | --- | --- | --- |
| Q401 | Hours worked per day | .........hours |  |
| Q402 | Hours worked per week | .........hours |  |
| Q403 | Regular health and safety supervision | 1. Yes 2. No | If no skip to 406 |
| Q404 | Did you receive any form of Formal training before joining construction? | 1.Yes 2.No |  |
| Q405 | If yes, What type of training did you receive? | 1. Apprentice 2.Vocational School 3. Others Specify ------------------------ |  |
| Q406 | Are machines you are working with always guarded or installed With safety devices? | 1. Yes 2. No |  |
| Q407 | Are machines you are working withal ways maintained Immediately when old or unsafe? | 1. Yes 2. No |  |

**Part-V- Workers behavior and characteristic**

| SN | Questions | Possible Response | Skips |
| --- | --- | --- | --- |
| Q501 | Do you smoke? | 1.Yes 2.No | If no skip to Q503 |
| Q502 | If yes for Q501, how often? | 1.Every day 2.1-3 stick days/ week 3.Ocassionally |  |
| Q503 | Do you drink alcohol? | 1.Yes 2.No | If no skip to Q505 |
| Q504 | If yes to Q503, how Often? | 1. Every day 2.1-5 days/wk. 3.Ocassionally |  |
| Q505 | Do you chew chat? | 1.Yes 2.No |  |
| Q506 | If yes to Q504, how Often | 1. Every day 2.1-3 days/wk. 3.Ocassionally |  |

Questionnaire ID: __________

That is the end of our questionnaire. Thank you very much for taking time to answer these Questions.

## Annex 3. Qualitative part data collection tool

**Interviewee guide for Focus group discussion**

**For workers**

1. What is the common occupational health problem that arises in your working place?
2. What are the major factors for the occurrence of those health problems?
   1. What kind of mechanism that you use so as to minimize those health problems that occur during the time of work?
   2. What methods can you explain that will reduce occupational health problems that will rise in your working environment?
3. In your opinion what are the relevance of health and safety system?
   1. How do you think these health and safety system protect workers from injuries?
4. Do all workers use PPE?
   1. If no why do you think the reason for not using PPE?
5. What type of safety measures do you expect from the employer? / Government?

Thank you very much for taking time to answer these Questions!!!

**For Governmental stakeholders**

1. What is the common occupational health problem that arises in the organizations that you inspecting?

1.1. What do you think the reason for these occupational health problems?

1.2. What methods can you explain that will reduce occupational health problems that will rise in your working environment?

2. In building construction industries is there occupational health and safety system?

2.1 Do you know building construction draft policy?

2.2 What do you think rules and regulations about construction?

3. In your opinion what are the relevance of health and safety system?

3.1. How do you think these health and safety system protect workers from injuries?

4. Do you inspect workers to use PPE properly?

4.1. If no why do you think the reason for not using PPE?

5. What type of safety measures do you expect from the employees (employer)?

Thank you very much for taking time to answer these Questions!!!

**For Contractors**

1. What do you think the reason for occupational health problems occurred on workers?

1.1. What do you think the reason for such occupational health problems?

1.2. What methods can you explain that will reduce occupational health problems that will rise in your working environment?

2. In building construction industries is there occupational health and safety system?

2.1 Do you have trade union? What about safety committee?

2.2 Do you know building construction draft policy?

2.3 What do you think rules and regulations about construction?

3. What is the relevance of health and safety system in your construction industries?

4. Do you inspect workers to use PPE properly?

4.1. If no why do you think the reason for not using PPE?

5. What type of safety measures do you expect from the employees?

5.1. What type of safety measures do you expect from the government?

Thank you very much for taking time to answer these Questions!!!
